# Fluorine-18-Fluorodeoxyglucose PET/CT Proved Valvular Endocarditis in a Native Valve Patient

**DOI:** 10.7759/cureus.48315

**Published:** 2023-11-05

**Authors:** Pavithra Dhanasekaran, Rajesh Gurunathan, Tejaswini Chavan

**Affiliations:** 1 Geriatric Medicine, Royal Stoke University Hospital, University Hospitals of North Midlands, Stoke-on-Trent, GBR; 2 Respiratory Medicine, Nottingham City Hospital, Nottingham University Hospitals NHS Trust, Nottingham, GBR

**Keywords:** transesophageal echocardiography in infective endocarditis, 18f-fdg pet ct to diagnose infective endocarditis, anticoagulation in infective endocarditis, pet-ct with 18f-fdg, mitral endocarditis, atypical infective endocarditis, native valve endocarditis, 18f-fdg pet/ct, 18f-fdg pet ct in endocarditis

## Abstract

A 79-year-old woman with a background history of hypertension, diabetes mellitus, hypercholesterolemia, alcoholic liver disease, and osteoporosis presented to the hospital with fever and confusion. Based on the initial investigations, it was challenging to identify the primary source of infection. Positive group G streptococcus on blood cultures raised the suspicion of uncommon infective endocarditis (IE). Two transthoracic echocardiographic studies were performed a week apart, and both were inconclusive. She was also diagnosed with atrial fibrillation, and the decision for anticoagulation became difficult as the clinical suspicion for IE was very high. Fluorine-18-fluorodeoxyglucose (18F-FDG) PET/CT confirmed the diagnosis of native valve endocarditis (NVE), and the possible complication related to anticoagulation was prevented. Though 18F-FDG PET/CT is commonly used to evaluate prosthetic valve endocarditis (PVE) and IE related to cardiac devices and catheters, its role and applicability to evaluate challenging cases of NVE could be entertained.

## Introduction

Infective endocarditis (IE) is a rare infectious disease affecting the endocardial surface of the heart [1]. It typically involves cardiac valves and devices inserted into the heart and is caused by virulent microorganisms [1,2]. Early diagnosis and treatment are critical, as IE can lead to a broad spectrum of intracardiac and extracardiac complications, which in turn cause significant morbidity and mortality [2]. IE is diagnosed based on modified Duke's criteria and depends on characteristic clinical signs and symptoms, positive blood cultures, and positive echocardiographic studies [2,3]. However, many patients may not present with classical history and physical examination findings, and diagnosing IE may be problematic [3,4]. The European Society of Cardiology (ESC) 2015 guidelines proposed other supplementary imaging techniques, including fluorine-18-fluorodeoxyglucose (18F-FDG) PET/CT, to improve the sensitivity of the modified Duke's criteria, mainly when the initial echocardiographic and microbiological studies are inconclusive with high clinical suspicion of IE [5]. These additional imaging modalities have been widely accepted to diagnose prosthetic valve endocarditis (PVE) and cardiac device-related IE (CDRIE) [5]. However, the evidence of its use in diagnosing native valve endocarditis (NVE) is limited [5]. This report describes not only the exciting case presentation of a frail elderly patient with native valves who was successfully diagnosed and treated for NVE with the help of 18F-FDG PET/CT but also the broad applicability of 18F-FDG PET/CT to identify IE and related complications in complex cases. 

## Case presentation

A 79-year-old lady with a background of multiple co-morbidities, including type 2 diabetes mellitus, essential hypertension, hypercholesterolemia, alcoholic liver disease, and osteoporosis, presented to the emergency department with a high-grade fever and mild confusion. The patient denied chest pain, shortness of breath, dizziness, syncope, and headache. Cardiovascular examination was unremarkable, and no cardiac murmur was heard on auscultation. On respiratory examination, reduced air entry was noted on the right side, but no added crackles or wheeze. Her electrocardiography (ECG) showed atrial fibrillation, which was a new diagnosis. Laboratory investigations are presented in Table 1. C-reactive protein (CRP) level and white blood cell count (WCC) were elevated. Blood culture was found to be positive for gram-positive group G streptococcus. The microbiology team advised treatment with intravenous benzylpenicillin 1.2 g four times a day and also advised ruling out the uncommon sources of infection, including discitis and endocarditis.

**Table 1:** Biochemical and hematological investigations

Name and type of investigation	Result	Normal range
Biochemistry		
Serum sodium	129 mEq/L	135 - 145 mEq/L
Serum potassium	3.3 mEq/L	3.5 - 5.5 mEq/L
Urea	9.1 mmol/L	2.5 - 7.8 mmol/L
Creatinine	97 mmol/L	59 - 104 mmol/L
Albumin	26 g/L	35 - 50 g/L
Alkaline phosphatase	85 IU/L	30 - 130 IU/L
Alanine transaminase	68 IU/L	10 - 40 IU/L
Total bilirubin	25 mmol/L	<21 mmol/L
C-reactive protein	228 mg/L	<4 mg/L
Adjusted calcium	2.38 mmol/L	2.1 - 2.6 mmol/L
Hematology		
Hemoglobin	115 g/L	120 - 160 g/L
Platelets	123 × 10^9^/L	150 - 400 × 10^9^/L
Mean corpuscular volume	94.2 fL	83 - 100 fL
Neutrophile count	12.5 × 10^9^/L	1.5 - 8.0 × 10^9^/L
White blood cell count	14.9 × 10^9^/L	4 - 11 × 10^9^/L

Transthoracic echocardiography (TTE) was performed, which showed heavily calcified mitral annulus and associated thickening on the undersurface of the posterior mitral valve leaflet with independent motion seen in some views. However, it could not exclude valvular endocarditis. The cardiology team advised to repeat TTE in a week's time. MRI of the whole spine showed features including multilevel disc degeneration, facet joint osteoarthritis, and long-standing osteoporotic compression fracture of T12. The intervertebral discs at lumbar vertebrae L2/L3 and L3/L4 levels were noted to have a fluid signal. The radiologist reported that the fluid signal at L3/L4 could be degenerative; however, the fluid signal at L2/L3 could be infectious. No other intraspinal or paraspinal fluid collections were noted. The spine team advised that there was no indication for any surgical intervention and to continue the antibiotic as per microbiology advice, as the patient was responding to the treatment. The second TTE was performed one week after the previous study, which showed a thickened echo bright mass associated with the posterior mitral valve leaflet and appeared more significant than the last TTE. These features suggested the possibility of leaflet vegetation; however, it was also concluded as inconclusive to diagnose IE.

**Figure 1:**
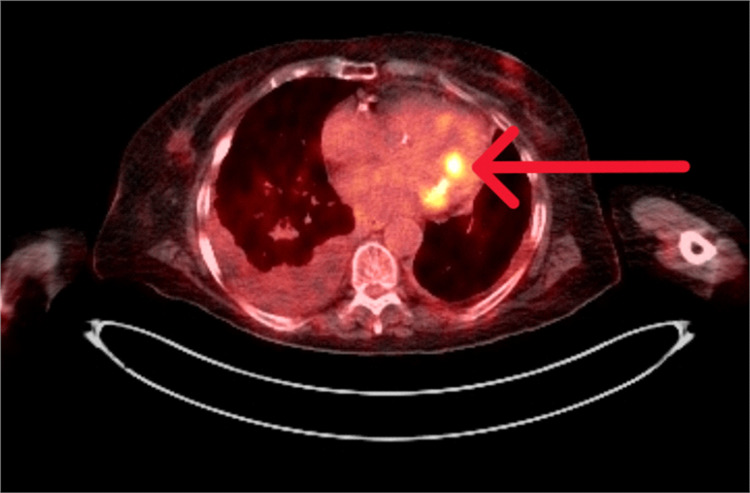
Cross-sectional image of 18F-FDG PET/CT showing increased FDG uptake of mitral valve 18F-FDG, fluorine-18-fluorodeoxyglucose; FDG, fluorodeoxyglucose

**Figure 2:**
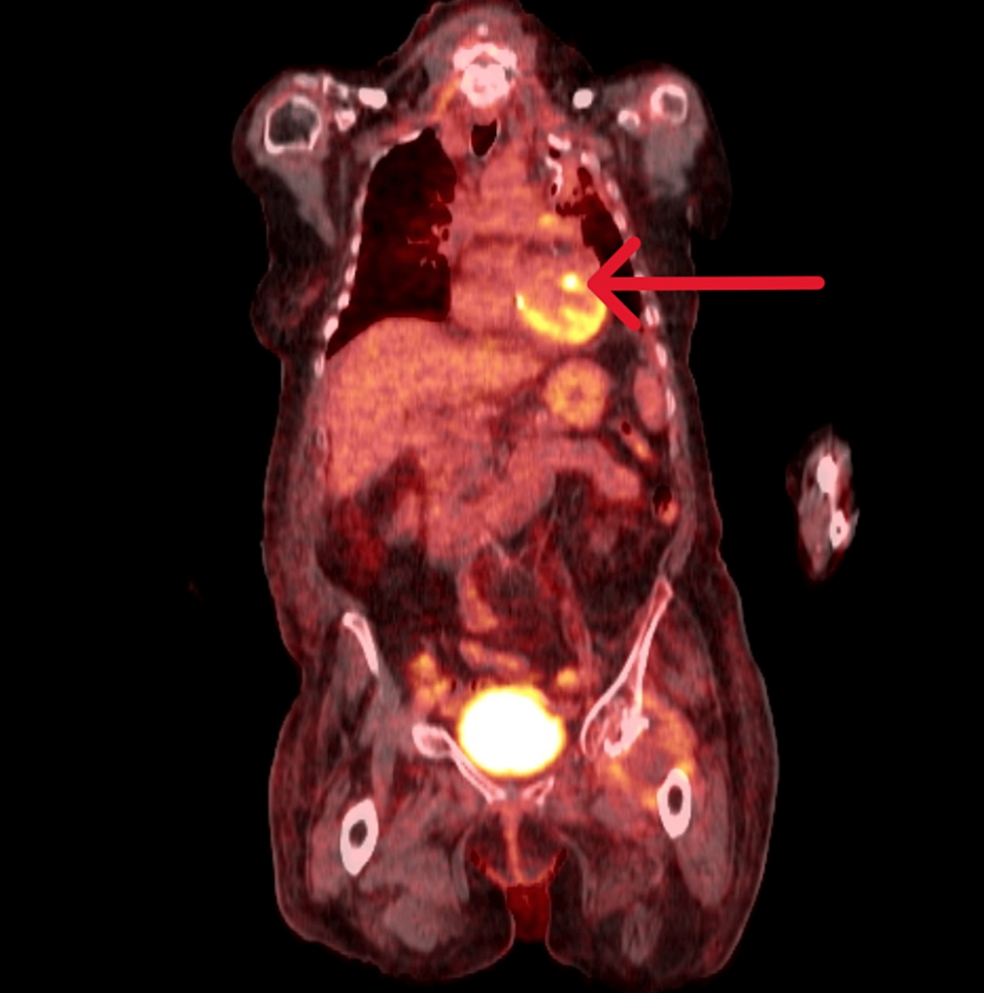
Coronal section of 18F-FDG PET/CT showing increased FDG uptake of mitral valve 18F-FDG, fluorine-18-fluorodeoxyglucose; FDG, fluorodeoxyglucose

In the meantime, the levels of CRP again started rising, and she had a temperature spike of 40.2°C. The microbiology team advised increasing the frequency of benzylpenicillin to 1.2 g every four hours, with which she showed a dramatic clinical response. She scored four on the CHA2DS2-VASc score for atrial fibrillation stroke risk assessment. However, she was not anticoagulated because of the possible risk of intracranial bleeding if the patient had IE. She was referred to Endocarditis and Spine Multidisciplinary Teams (MDT). As both the TTE studies were inconclusive in diagnosing IE and the MRI scan of the spine showed a suspicious fluid signal within the discs at L2/L3, the MDTs advised performing 18-F FDG PET/CT to ensure whether the patient had discitis or endocarditis. 18-F-FDG PET/CT showed multiple findings, including densely calcified mitral valve annulus, an exophytic focus of increased FDG uptake along the anterolateral aspect of the mitral valve (Figures 1-2) projecting into the lumen of the left ventricle probably vegetation supportive of valvular endocarditis, no FDG avid cervical or mediastinal lymphadenopathy, no metabolic activity seen in the liver or other solid abdominal organs, no intra-abdominal collection, no abnormal FDG uptake seen in the intervertebral disc spaces, and no metabolic evidence of active discitis.

Based on the above clinical and investigatory findings, differential diagnoses considered were (1) IE: persistent fever of unknown origin, positive group G streptococcus blood culture, and thickened echo bright mass associated with the posterior mitral valve, which increased in size when compared to that of the previous study and increased FDG uptake of the mitral valve on 18F-FDG PET/CT; (2) discitis: fluid signal noted at L2/L3 and L3/L4 levels on MRI whole spine; however, 18F-FDG PET/CT strongly favored the diagnosis of valvular IE and ruled out the possibility of discitis. 

During her hospital stay, she was initially treated with an empirical antibiotic (co-amoxiclav) and IV fluids. When the blood culture tested positive for gram-positive streptococci, the antibiotic was switched to benzylpenicillin. Group G streptococcus was isolated from the blood culture, and the treatment was continued with benzylpenicillin. After treating her for one week, she had a temperature spike and raised CRP levels. The frequency of benzylpenicillin was increased to four hours, with which she showed a dramatic clinical response. As 18F-FDG PET/CT confirmed the diagnosis of valvular endocarditis, she completed a four-week course of antibiotic therapy. She was at high risk of developing thromboembolic events from atrial fibrillation as she scored four on CHA2DS2-VASc; however, she was not anticoagulated during the antibiotic therapy, given the high risk of intracranial hemorrhage associated with septic emboli. After antibiotic treatment, she was given lower molecular weight heparin for two weeks and then switched to a non-vitamin K oral anticoagulant (apixaban). Her blood investigations and observations remained stable, and she was discharged with a plan to repeat TTE in four to six weeks and review the patient in the outpatient Cardiology Clinic. 

## Discussion

IE is a rare infectious disease with an estimated incidence of five to 15 cases per 100,000 people [1]. It is caused by various microorganisms, mainly by gram-positive streptococci, staphylococci, and enterococci, and less commonly by HACEK organisms (Haemophilus, Actinobacillus, Cardiobacterium, Eikenella, and Kingella) [1-3]. If not diagnosed and treated early, IE can lead to significant morbidity and mortality [2]. Because of its variable presentation and potential to rapidly progress to cause lethal complications, clinical history and detained physical examination are critical for early identification of IE and its related complications, thereby treating it effectively [2]. 

The progression of the disease requires antecedent endocardial injury and a subsequent phase of bacteremia [2]. The injured endocardium allows platelet aggregation and forms sterile thrombotic vegetation, which is then colonized by the microorganism [2]. IE may present with a spectrum of signs and symptoms; fever is the most commonly reported symptom in almost 95% of cases, and the less common symptoms include chills, excessive tiredness, malaise, anorexia, and headache. [2]. People with valvular insufficiency may also present with other cardiopulmonary symptoms, including chest pain, orthopnea, paroxysmal nocturnal dyspnea, reduced exercise tolerance, and signs of underlying infection [2]. Almost one-third of the cases reported having symptoms of heart failure secondary to acute valvular incompetence [2]. Valvular regurgitation can cause atrial enlargement and stretch, which subsequently cause atrial fibrillation and other dysrhythmias [2]. The cardiorespiratory examination may reveal a new or worsening cardiac murmur, characteristic of most cases of IE [2]. Extracardiac signs, including Osler's nodes, splinter hemorrhage, Janeway lesions of the eye, Roth spots, petechiae, and clubbing, were reported to be associated with IE in a small group of case patients [2].

Intravenous drug abuse, degenerative valvular disease, prosthetic valve placement, implanted cardiac devices and catheters, diabetes, rheumatic heart disease, and immunosuppression are commonly reported risk factors for developing IE [2]. Many intracardiac and extracardiac complications have been reported to be associated with IE [2,3]. Almost any distant organ can be affected because of the risk of lodgement of septic emboli through circulation [2,3]. Extracardiac complications usually arise from an embolic origin, of which ischemic stroke is the most common neurological complication, and others include intracranial hemorrhage and abscesses, meningitis, and infective aneurysm [2,3]. Right-sided vegetation, which is quite common among intravenous drug users, can lead to pulmonary abscesses, pneumonia, emphysema, and pulmonary infarcts [2,5-6]. Other than embolic complications, immune-mediated glomerulonephritis cases have also been reported [2]. The frequency of complications mainly depends on the pathogen caused, the disease's duration before antibiotic therapy, and the choice of treatment provided [3].

IE has been traditionally diagnosed based on Duke's criteria, which was initially set out in 1994 and then modified in 2000 because of its lower specificity to analyze blood culture-negative IE and prosthetic valve-related IE and to reduce the size of broadly classified possible IE groups [3]. The components of the modified Duke's criteria include two major criteria and five minor criteria [3]. The first major criterion involves confirmation of bacteremia, precisely two positive blood cultures for typical organisms, persistently positive blood cultures for other microorganisms, and positive blood culture for Coxiella burnetii or anti-phase I immunoglobulin G antibody titer ≥1:800 to diagnosed cases of Q-fever IE [3]. The second major criterion involves evidence of endocardial involvement [3]. The American Heart Association (AHA) recommends TTE for all cases of suspected IE, and transesophageal echocardiography (TEE) needs to be considered, if TTE is negative and suspicion of IE is high [3]. The minor criteria include predisposing risk factors, fever, the presence of vascular and immunologic phenomena, and evidence of bacteremia that does not satisfy the major criterion [3]. The diagnosis of IE needs the satisfaction of either two major criteria or one major and three minor or five minor criteria [3]. Although IE has been widely studied over the years, many people are reported to have non-specific atypical symptoms, sometimes making the diagnosis of IE challenging for clinicians [3,4]. 

Because of the embolic risk associated with IE, using anticoagulants and antiplatelet therapy in cases with IE is extensively studied [6]. Various studies recommend using them only after treating IE with antibiotics for the appropriate duration [6]. The ESC 2015 recommended that the decision for anticoagulation in IE be made individually, weighing the risks and benefits involved [6]. 

The ECS 2015 guidelines also support the use of TEE when TTE is either normal or inconclusive, with high suspicion of IE [6]. TTE is considered the first-line imaging technique in all the suspected cases of IE; however, underlying valve disease may influence the diagnostic accuracy of TTE when a myxomatous mitral valve or sclerotic or calcified valves or little vegetation <3 mm is present [7]. TEE is highly sensitive to identifying PVE and NVE, but it is immensely challenging to identify IE affecting intracardiac devices [7]. Various studies suggest that the sensitivity of TTE ranges between 40% and 63%, whereas TEE ranges between 90% and 100% [7]. Echocardiographic studies demonstrate highly variable diagnostic accuracy, may provide false positive results, and sometimes, it is challenging to differentiate vegetation from thrombi, cusp prolapse, chordal rupture, myxomatous or degenerative valve disease, non-bacterial vegetation, and normal anatomical variations [8]. 

In 2015, the ESC included additional imaging modalities, including 18F-FDG PET/CT, leucocyte labeled SPECT/CT, and cardiac CT, to improve the sensitivity of the modified Duke's criteria and thereby diagnosing cardiac involvement and embolic events, especially when the echocardiographic studies are inconclusive [9]. 18F-FDG PET/CT is an important tool for evaluating IE, and its sensitivity is variable: high for diagnosing PVE and CDRIE pocket infections and low for NVE and CDRIE-lead infection [9]. The evidence for using 18F-FDG PET/CT in NVE is limited because of its lower sensitivity; however, the specificity of the same is very high, and it is recommended in NVE to identify the disseminated source of infection and, if diagnosed, is strongly suggestive of a definite diagnosis, which gives 18F-FDG PET/CT additional suitability in evaluating challenging cases [9]. Various factors determine the overall diagnostic accuracy of 18F-FDG PET/CT, including the presence of the different cardiac prosthetic materials, the patient preparation with a high fat-low carbohydrate diet, scan acquisition, image quality, recent cardiac surgery, and the duration of antibiotic treatment before the scan [10]. 

The use of 18F-FDG PET/CT to diagnose valvular endocarditis has been recommended by a limited number of studies, most of which suggested the need for proper standardization procedures that must be followed to obtain better diagnostic accuracy [11,12]. The promising benefits of 18F-FDG PET/CT in the evaluation of IE reported are its ability to identify intracardiac infection, provide additional information about embolization and seeding of disease to distant organs, and provide evidence of involvement of the foreign body [11,12]. Considering all these benefits, 18F-FDG PET/CT offers invaluable information that may significantly impact the diagnosis, decision-making, and treatment planning, and the role of 18F-FDG PET/CT in challenging cases or its additional ability to provide additional diagnostic information is not questionable [12].

## Conclusions

This is a case of NVE that has been a diagnostic challenge. After two inconclusive echocardiographic studies, 18F-FDG PET/CT confirmed the diagnosis of valvular endocarditis and ruled out other sources of infection. It is an effective imaging modality in evaluating IE, especially when the initial microbiologic and echocardiographic studies are inconclusive with strong clinical suspicion. Furthermore, it can identify the metastasis of the infection, detect inflammation before the structural changes happen, and recognize the foreign body involved, all with which it possesses a wide range of applicability in clinical practice to diagnose complex cases of both PVE and NVE. 18F-FDG PET/CT can be considered to diagnose challenging cases of IE, even in a native valve patient. Better patient preparation protocols and technical advancements in scan acquisition may improve its sensitivity and usability in future studies.
